# Supplementary material for: G protein–coupled receptor kinase 3 couples atypical chemokine receptor 4 independent of G proteins
Source: Mol Pharmacol. 2026 May 12;108(5-6):100129. doi: 10.1016/j.molpha.2026.100129 (PMC13494237; doi:10.1016/j.molpha.2026.100129)
Supplement: Supplementary file 1 [file mmc1.docx]

**Supplemental information**

**GPCR kinase 3 couples atypical chemokine receptor 4 independent of G proteins**

Thomas D. Lamme, Isabel B. Sánchez-Arroyo, Martine J. Smit, Christopher T. Schafer^1^.

Division of Medicinal Chemistry, Faculty of Science, Vrije Universiteit Amsterdam, De Boelelaan 1108, 1081 HZ Amsterdam, The Netherlands.

^1^To whom correspondence should be addressed: Christopher T. Schafer, [c.t.schafer@vu.nl](mailto:c.t.schafer@vu.nl)

**Supplemental Information**

**Supplemental Figure 1. ACKR4 expression in HEK293 parental cells and GRK knockouts.** Total expression of ACKR4-RlucII measured by luminescence counts (410-80 nm) in HEK293, ΔGRK2/3, ΔGRK5/6, and ΔQ cells. Values represent the mean ± SD of three independent experiments performed in triplicate normalized to WT luminescence. Statistical significance compared to parental cells was determined by one-way Brown–Forsythe and Welch ANOVA followed by a Dunnett's T3 multiple comparisons test. *p < 0.05

**Supplemental Figure 2. Evaluation of GRK2/3 protein levels in the addback experiments presented in Fig. 3.** Western blots reporting GRK2 (A) and GRK3 (B) protein levels in ΔQ cells with overexpression of pcDNA3.1, WT GRK, R587Q GRK, or R587Q/D110A GRK. Quantification of band intensity over control bands (β-actin) of GRK2 (C) or GRK3 (D).

**Supplemental Figure 3. ACKR4 expression levels of DE/A mutants in HEK293 cells.** Total expression of ACKR4-RlucII measured by luminescence counts (410-80 nm). Values represent the mean ± SD of three independent experiments performed in triplicate normalized to WT luminescence. Statistical significance compared to WT ACKR4 expression was determined by one-way Brown–Forsythe and Welch ANOVA followed by a Dunnett's T3 multiple comparisons test. *p < 0.05

**Supplemental Figure 4. Pairwise correlations between GRK3 recruitment, β-arr2 recruitment, and CCL25 uptake.** Correlation analysis of (A) GRK3 recruitment and β-arr2 recruitment, (B) β-arr2 recruitment and uptake, (C) GRK3 recruitment and uptake, and (D) constitutive β-arr2 recruitment and uptake (from **Fig.6**). R^2^ and corresponding p values from Pearson correlation are shown in each panel.

**Supplemental Figure 5. ACKR4 surface expression levels of DE/A mutants in HEK293 cells.** Surface expression of untagged ACKR4 measured by flow cytometry. Values represent the mean ± SD of three independent experiments performed in triplicate normalized to WT mean fluorescence intensity (MFI). Statistical significance was determined by one-way Brown–Forsythe and Welch ANOVA followed by a Dunnett's T3 multiple comparisons test. *p < 0.05, **p<0.001

**Supplemental Figure 6. Predicted ACKR4 C-terminal orientation near the kinase domain.** (A) Alphafold3.0 model of the CCL25-ACKR4-GRK3 complex with predicted local distance difference test (pLDDT) per residue. (Inset) C-terminus of ACKR4 inside the pocket of GRK3. (B) PAE plot of CCL25 [A], ACKR4 [B], GRK3 [C]. Plots are generated by PAE Viewer ^1^.

**Supplemental Figure 7. GRK3 expression levels in HEK293 cells.** Total expression of GRK3-Nluc measured by luminescence counts (475-30 nm). Values represent the mean ± SD of three independent experiments (per GPCR) performed in triplicate normalized to WT luminescence. Statistical significance comparing the expression of mutant GRKs to WT GRK3 was determined by one-way Brown–Forsythe and Welch ANOVA followed by a Dunnett's T3 multiple comparisons test. *p < 0.001, and **p < 0.0001.

**Supplemental Figure 8. (KD-)GRK3 expression levels in HEK293 cells.** Total expression of GRK3-Nluc measured by luminescence counts (475-30 nm). Bars represent the mean ± SD of three independent experiments (points) performed in triplicate normalized to WT luminescence.

**Supplemental Figure 9. ST/A mutation impairs ACKR4-mediated GRK3 localization towards the membrane.** (A) GRK3 localization towards the membrane measured as bystander BRET between GRK3-Nluc and mV-CAAX with co-expression of untagged WT ACKR4 and ACKR4 ST/A (S330A/S338A/T342A/T345A/S346A/T347A/S349A). Transfected DNA for ACKR4 ST/A was increased to match surface expression of WT. (B) Quantification by AUC. (C) Surface expression of untagged ACKR4 measured by flow cytometry. Values represent the mean ± SD of three independent experiments performed in triplicate. Statistical significance was determined by one-way Brown–Forsythe and Welch ANOVA followed by a Dunnett's T3 multiple comparisons test (basal). *p< 0.05, **p < 0.001.

**Supplemental Table 1. Statistics**

| **Fig.** | **Condition** | **Emax** | | **Bar graph** | | | **Statistical test** |
| --- | --- | --- | --- | --- | --- | --- | --- |
|  |  | **P-value** | **Value ± SD/CI*** | **P-value** | **Value ± SD** | |  |
| **1c** | HEK P | N.A | 86.33 **±** 5.08* | N.A. | | | Extra sum-of-squares F test |
|  | ΔGRK2/3 | <0.0001 | 28.13 **±** 2.89* |  |  |  |  |
|  | ΔGRK5/6 | <0.0001 | 62.94 **±** 4.91* |  |  |  |  |
|  | ΔQ | <0.0001 | 3.37 ± 0.54* |  |  |  |  |
| **2b-L** | - | N.A. | 0.122 ± 0.014 | N.A. | | | t-test |
|  | +GRK3-CT | 0.43 | 0.145 ± 0.038 |  |  |  |  |
| **2c-L** | - | N.A. | 0.044 ± 0.0098 | N.A. | | | t-test |
|  | +GRK3-CT | 0.019 | 0.013 ± 0.0039 |  |  |  |  |
| **2d-L** | - | N.A. | 0.12 ± 0.013 | N.A. | | | t-test |
|  | +GRK3-CT | 0.0096 | 0.056 ± 0.0038 |  |  |  |  |
| **2e-L** | - | N.A. | 0.059 ± 0.010 | N.A. | | | t-test |
|  | +GRK3-CT | 0.017 | 0.0090 ± 0.016 |  |  |  |  |
| **2b-R** | - | N.A. | N.A. | N.A. | | | Extra sum-of-squares F test |
|  | +GRK3-CT | N.A. | N.A. |  |  |  |  |
|  | +Gby | N.A. | N.A. |  |  |  |  |
| **2c-R** | - | N.A. | 98.02 ± 4.58 | N.A. | | | Extra sum-of-squares F test |
|  | +GRK3-CT | <0.0001 | 63.69 ± 3.43 |  |  |  |  |
|  | +Gby | <0.0001 | 123.7 ± 1.80 |  |  |  |  |
| **2d-R** | - | N.A. | 93.49 ± 4.30 | N.A. | | | Extra sum-of-squares F test |
|  | +GRK3-CT | <0.0001 | 65.71 ± 4.01 |  |  |  |  |
|  | +Gby | N.A. | N.A. |  |  |  |  |
| **2e-R** | - | N.A. | 101.2 ± 2.21 | N.A. | | | Extra sum-of-squares F test |
|  | +GRK3-CT | <0.0001 | 89.03 ± 2.97 |  |  |  |  |
|  | +Gby | N.A. | N.A. |  |  |  |  |
| **3b-L** | ΔQ | N.A. | | N.A. | | 0.49 ± 0.05 | Browns-Forsythe & Welch ANOVA |
|  | ΔQ+GRK2 |  |  | 0.004 | | 2.05 ± 0.09 |  |
|  | ΔQ+GRK2 R587Q |  |  | 0.017 | | 0.90 ± 0.09 |  |
|  | ΔQ+GRK3 |  |  | 0.004 | | 2.11 ± 0.10 |  |
|  | ΔQ+GRK3  R587Q |  |  | 0.0023 | | 0.90 ± 0.06 |  |
| **3c-L** | ΔQ | N.A. | | N.A. | | 0.91 ± 0.04 | Browns-Forsythe & Welch ANOVA |
|  | ΔQ+GRK2 |  |  | 0.040 | | 1.67 ± 0.17 |  |
|  | ΔQ+GRK2 R587Q |  |  | 0.96 | | 0.92 ± 0.04 |  |
|  | ΔQ+GRK3 |  |  | 0.002 | | 1.78 ± 0.09 |  |
|  | ΔQ+GRK3  R587Q |  |  | 0.95 | | 0.92 ± 0.05 |  |
| **3d-L** | ΔQ | N.A. | | N.A. | | N.A. | Browns-Forsythe & Welch ANOVA |
|  | ΔQ+GRK2 |  |  | N.A. | | N.A. |  |
|  | ΔQ+GRK2 R587Q |  |  | N.A. | | N.A. |  |
|  | ΔQ+GRK3 |  |  | N.A. | | N.A. |  |
|  | ΔQ+GRK3  R587Q |  |  | N.A. | | N.A. |  |
| **3f-L** | ΔQ | N.A. | | N.A. | | 0.47 ± 0.02 | Browns-Forsythe & Welch ANOVA |
|  | ΔQ+GRK2 |  |  | 0.0007 | | 1.95 ± 0.09 |  |
|  | ΔQ+GRK2 R587Q/D110A |  |  | 0.003 | | 0.90 ± 0.04 |  |
|  | ΔQ+GRK3 |  |  | 0.0001 | | 1.94 ± 0.05 |  |
|  | ΔQ+GRK3  R587Q/D110A |  |  | 0.018 | | 0.74 ± 0.08 |  |
| **3g-L** | ΔQ | N.A. | | N.A. | | N.A. | Browns-Forsythe & Welch ANOVA |
|  | ΔQ+GRK2 |  |  | N.A. | | N.A. |  |
|  | ΔQ+GRK2 R587Q/D110A |  |  | N.A. | | N.A. |  |
|  | ΔQ+GRK3 |  |  | N.A. | | N.A. |  |
|  | ΔQ+GRK3  R587Q/D110A |  |  | N.A. | | N.A. |  |
| **3b-R** | ΔQ | N.A. | N.A. | N.A. | | | Extra sum-of-squares F test |
|  | ΔQ+GRK2 | N.A. | 62.05 ± 4.73 |  |  |  |  |
|  | ΔQ+GRK2 R587Q | <0.0001 | 90.48 ± 5.55 |  |  |  |  |
|  | ΔQ+GRK3 | N.A. | 60.79 ± 5.90 |  |  |  |  |
|  | ΔQ+GRK3  R587Q | <0.0001 | 102.4 ± 5.11 |  |  |  |  |
| **3c-R** | ΔQ | N.A. | N.A. | N.A. | | | Extra sum-of-squares F test |
|  | ΔQ+GRK2 | N.A. | 88.59 ± 4.02 |  |  |  |  |
|  | ΔQ+GRK2 R587Q | <0.0001 | 38.41 ± 2.97 |  |  |  |  |
|  | ΔQ+GRK3 | N.A. | 83.51 ± 3.72 |  |  |  |  |
|  | ΔQ+GRK3  R587Q | <0.0001 | 43.33 ± 2.01 |  |  |  |  |
| **3d-R** | ΔQ | N.A. | N.A. | N.A. | | | Extra sum-of-squares F test |
|  | ΔQ+GRK2 | N.A. | 77.57 ± 5.87 |  |  |  |  |
|  | ΔQ+GRK2 R587Q | <0.0001 | 25.90 ± 2.95 |  |  |  |  |
|  | ΔQ+GRK3 | N.A. | 78.57 ± 5.12 |  |  |  |  |
|  | ΔQ+GRK3  R587Q | <0.0001 | 41.55 ± 2.53 |  |  |  |  |
| **3f-R** | ΔQ | N.A. | N.A. | N.A. | | | Extra sum-of-squares F test |
|  | ΔQ+GRK2 | N.A. | N.A. |  |  |  |  |
|  | ΔQ+GRK2 R587Q/D110A | N.A. | N.A. |  |  |  |  |
|  | ΔQ+GRK3 | N.A. | N.A. |  |  |  |  |
|  | ΔQ+GRK3  R587Q/D110A | N.A. | N.A. |  |  |  |  |
| **3g-R** | ΔQ | N.A. | N.A. | N.A. | | | Extra sum-of-squares F test |
|  | ΔQ+GRK2 | N.A. | 86.65 ± 4.34 |  |  |  |  |
|  | ΔQ+GRK2 R587Q/D110A | <0.0001 | 51.73 ± 2.56 |  |  |  |  |
|  | ΔQ+GRK3 | N.A. | 88.72 ± 4.50 |  |  |  |  |
|  | ΔQ+GRK3  R587Q/D110A | <0.0001 | 50.85 ± 3.65 |  |  |  |  |
| **4b** | ΔQ | N.A. | | N.A. | | 0.86 ± 0.04 | Browns-Forsythe & Welch ANOVA |
|  | ΔQ+GRK2 |  |  | <0.0001 | | 1.72 ± 0.05 |  |
|  | ΔQ+GRK2 R587Q |  |  | 0.22 | | 0.95 ± 0.05 |  |
|  | ΔQ+GRK3 |  |  | 0.001 | | 1.95 ± 0.01 |  |
|  | ΔQ+GRK3  R587Q |  |  | 0.16 | | 0.95 ± 0.04 |  |
| **4c** | ΔQ | N.A. | N.A. | N.A. | | | Extra sum-of-squares F test |
|  | ΔQ+GRK2 | N.A. | N.A. |  |  |  |  |
|  | ΔQ+GRK2 R587Q | N.A. | N.A. |  |  |  |  |
|  | ΔQ+GRK3 | N.A. | N.A. |  |  |  |  |
|  | ΔQ+GRK3  R587Q | N.A. | N.A. |  |  |  |  |
| **6b** | WT | N.A. | | N.A. | | N.A. | Browns-Forsythe & Welch ANOVA |
|  | E332A/E333A |  |  | 0.001 | | 61.31 ± 2.2 |  |
|  | D337A/E339A |  |  | 0.003 | | 56.10 ± 4.4 |  |
|  | Proximal |  |  | 0.002 | | 50.87 ± 3.7 |  |
|  | Distal |  |  | 0.006 | | 62.47 ± 5.1 |  |
|  | Acidic_all |  |  | 0.003 | | 48.43 ± 5.1 |  |
| **6c** | WT | N.A. | 91.55 ± 5.51 | N.A. | | | Extra sum-of-squares F test |
|  | E332A/E333A | <0.0001 | 61.22 ± 3.37 |  |  |  |  |
|  | D337A/E339A | <0.0001 | 65.37 ± 3.84 |  |  |  |  |
|  | Proximal | <0.0001 | 43.55 ± 3.01 |  |  |  |  |
|  | Distal | N.A. | N.A. |  |  |  |  |
|  | Acidic_all | 0.0045 | 13.93 ± 2.91 |  |  |  |  |
| **6d** | WT | N.A. | 90.11 ± 3.62 | N.A. | | | Extra sum-of-squares F test |
|  | WT + Gβγ | <0.0001 | 71.72 ± 4.14 |  |  |  |  |
|  | Acidic_all | N.A. | 14.82 ± 1.62 |  |  |  |  |
|  | Acidic_all + Gβγ | <0.0001 | 44.41 ± 2,61 |  |  |  |  |
| **6e** | WT | N.A. | | N.A. | | 100 ± 12.3 | Browns-Forsythe & Welch ANOVA |
|  | E332A/E333A |  |  | 0.015 | | 38.5 ± 5.08 |  |
|  | D337A/E339A |  |  | 0.019 | | 37.4 ± 7.84 |  |
|  | Proximal |  |  | 0.003 | | 20.6 ± 8.55 |  |
|  | Distal |  |  | 1.00 | | 106.8 ± 31.3 |  |
|  | Acidic_all |  |  | 0.024 | | 18.1 ± 3.55 |  |
| **6f** | WT | N.A. | | N.A. | | N.A. | Browns-Forsythe & Welch ANOVA |
|  | E332A/E333A |  |  | 0.02 | | 31.8 ± 10.4 |  |
|  | D337A/E339A |  |  | 0.02 | | 42.7 ± 9.0 |  |
|  | Proximal |  |  | 0.002 | | 22.4 ± 3.5 |  |
|  | Distal |  |  | 0.04 | | 58.2 ± 9.3 |  |
|  | Acidic_all |  |  | 0.01 | | 21.3 ± 8.6 |  |
| **7b** | WT | ACKR4 | N.A. | N.A. | | N.A. | Browns-Forsythe & Welch ANOVA |
|  |  | CXCR4 |  |  |  | N.A. |  |
|  |  | CCR9 |  |  |  | N.A. |  |
|  | R226A | ACKR4 | N.A. | 0.057 | | 38.6 ± 8.5 | Browns-Forsythe & Welch ANOVA |
|  |  | CXCR4 |  |  |  | 67.2 ± 15.7 |  |
|  |  | CCR9 |  |  |  | 61.8 ± 10.8 |  |
|  | K230A | ACKR4 | N.A. | 0.057 | | 67.1 ± 7.0 | Browns-Forsythe & Welch ANOVA |
|  |  | CXCR4 |  |  |  | 72.2 ± 18.6 |  |
|  |  | CCR9 |  |  |  | 78.5 ± 10.4 |  |
|  | R316A | ACKR4 | N.A. | 0.37 | | 25.5 ± 5.3 | Browns-Forsythe & Welch ANOVA |
|  |  | CXCR4 |  |  |  | 16.3 ± 14.5 |  |
|  |  | CCR9 |  |  |  | 29.8 ± 9.5 |  |
|  | K319A | ACKR4 | N.A. | 0.55 | | 57.8 ± 19.3 | Browns-Forsythe & Welch ANOVA |
|  |  | CXCR4 |  |  |  | 83.5 ± 14.2 |  |
|  |  | CCR9 |  |  |  | 66.6 ± 39.7 |  |
|  | K345A | ACKR4 | N.A. | 0.15 | | 83.4 ± 2.5 | Browns-Forsythe & Welch ANOVA |
|  |  | CXCR4 |  |  |  | 78.2 ± 14.1 |  |
|  |  | CCR9 |  |  |  | 111.3 ± 24.1 |  |
|  | K364A | ACKR4 | N.A. | 0.46 | | 70.0 ± 11.5 | Browns-Forsythe & Welch ANOVA |
|  |  | CXCR4 |  |  |  | 74.7 ± 20.0 |  |
|  |  | CCR9 |  |  |  | 85.4 ± 8.2 |  |
|  | K383A | ACKR4 | N.A. | 0.44 | | 39.8 ± 23.0 | Browns-Forsythe & Welch ANOVA |
|  |  | CXCR4 |  |  |  | 51.3 ± 7.7 |  |
|  |  | CCR9 |  |  |  | 66.0 ± 30.3 |  |
| **8a** | WT | N.A. | | N.A. | | 100 ± 11.3 | t-test |
|  | KD-GRK3 |  |  | 0.002 | | 25.1 ± 7.0 |  |
| **8b** | WT | N.A. | | N.A. | | 100 ± 24.8 | t-test |
|  | KD-GRK3 |  |  | 0.24 | | 72.5 ± 24.5 |  |
| **8c** | WT | N.A. | | N.A. | | 100 ± 18.0 | t-test |
|  | KD-GRK3 |  |  | 0.49 | | 90.9 ± 8.7 |  |
| **8d** | WT | N.A. | | N.A. | | 100 ± 25.8 | t-test |
|  | KD-GRK3 |  |  | 0.12 | | 57.7 ± 26.5 |  |
| **S1** | HEK P | N.A. | | N.A. | | N.A. | Browns-Forsythe & Welch ANOVA |
|  | ΔGRK2/3 |  |  | 0.035 | | 57.4 ± 14.1 |  |
|  | ΔGRK5/6 |  |  | 0.049 | | 68.7 ± 12.4 |  |
|  | ΔQ |  |  | 0.012 | | 40.5 ± 11.7 |  |
| **S3** | WT | N.A. | | N.A. | | N.A. | Browns-Forsythe & Welch ANOVA |
|  | E332A/E333A |  |  | 0.26 | | 88.8 ± 12.3 |  |
|  | D337A/E339A |  |  | 0.27 | | 85.2 ± 17.2 |  |
|  | Proximal |  |  | 0.71 | | 95.4 ± 18.4 |  |
|  | Distal |  |  | 0.058 | | 72.4 ± 12.0 |  |
|  | All |  |  | 0.011 | | 43.9 ± 10.0 |  |
| **S5** | WT | N.A. | | N.A. | | N.A. | Browns-Forsythe & Welch ANOVA |
|  | E332A/E333A |  |  | 0.044 | | 38.0 ± 13.7 |  |
|  | D337A/E339A |  |  | 0.0077 | | 34.0 ± 6.0 |  |
|  | Proximal |  |  | 0.029 | | 24.5 ± 13.5 |  |
|  | Distal |  |  | 0.0097 | | 40.9 ± 6.1 |  |
|  | All |  |  | 0.0082 | | 18.1 ± 7.7 |  |
| **S7** | WT | N.A. | | N.A. | | N.A. | Browns-Forsythe & Welch ANOVA |
|  | R226A |  |  | <0.0001 | | 70.4 ± 13.6 |  |
|  | K230A |  |  | 0.0001 | | 67.2 ± 16.2 |  |
|  | R316A |  |  | <0.0001 | | 49.4 ± 10.3 |  |
|  | K319A |  |  | <0.0001 | | 65.1 ± 8.0 |  |
|  | K345A |  |  | 0.84 | | 100.7 ± 10.1 |  |
|  | K364A |  |  | 0.02 | | 86.8 ± 13.6 |  |
|  | K383A |  |  | 0.93 | | 100.8 ± 23.5 |  |
| **S8a** | WT | N.A. | | N.A. | | N.A. | t-test |
|  | KD-GRK3 |  |  | 0.58 | | 116.8 ± 44.1 |  |
| **S8b** | WT | N.A. | | N.A. | | N.A. | t-test |
|  | KD-GRK3 |  |  | 0.65 | | 123.3 ± 76.1 |  |
| **S8c** | WT | N.A. | | N.A. | | N.A. | t-test |
|  | KD-GRK3 |  |  | 0.65 | | 116.7 ± 54.3 |  |
| **S8d** | WT | N.A. | | N.A. | | N.A. | t-test |
|  | KD-GRK3 |  |  | 0.56 | | 121.8 ± 54.4 |  |
| **S9b** | WT | N.A. | | N.A. | | 100 ± 9.7 | t-test |
|  | ST/A |  |  | 0.0015 | | 36.9 ± 6.9 |  |
| **S9c** | WT | N.A. | | N.A. | | N.A. | t-test |
|  | ST/A |  |  | 0.049 | | 71.1 ± 8.9 |  |

**References**

1.	Elfmann, C. & Stülke, J. PAE viewer: a webserver for the interactive visualization of the predicted aligned error for multimer structure predictions and crosslinks. *Nucleic Acids Res* 51, W404 (2023).
